# Supplementary material for: Smoking drives an epigenetic memory of aberrant hematopoiesis
Source: medRxiv. 2026 May 21:2026.05.14.26353250. Preprint. [Version 1] doi: 10.64898/2026.05.14.26353250 (PMC13228793; doi:10.64898/2026.05.14.26353250)
Supplement: 1 [file NIHPP2026.05.14.26353250V1-supplement-1.pdf]

medRxiv preprint doi: https://doi.org/10.64898/2026.05.14.26353250; this version posted May 21, 2026. The copyright holder for this preprint (which was not certified by peer review) is the author/funder, who has granted medRxiv a license to display the preprint in perpetuity.
It is made available under a CC-BY 4.0 International license .

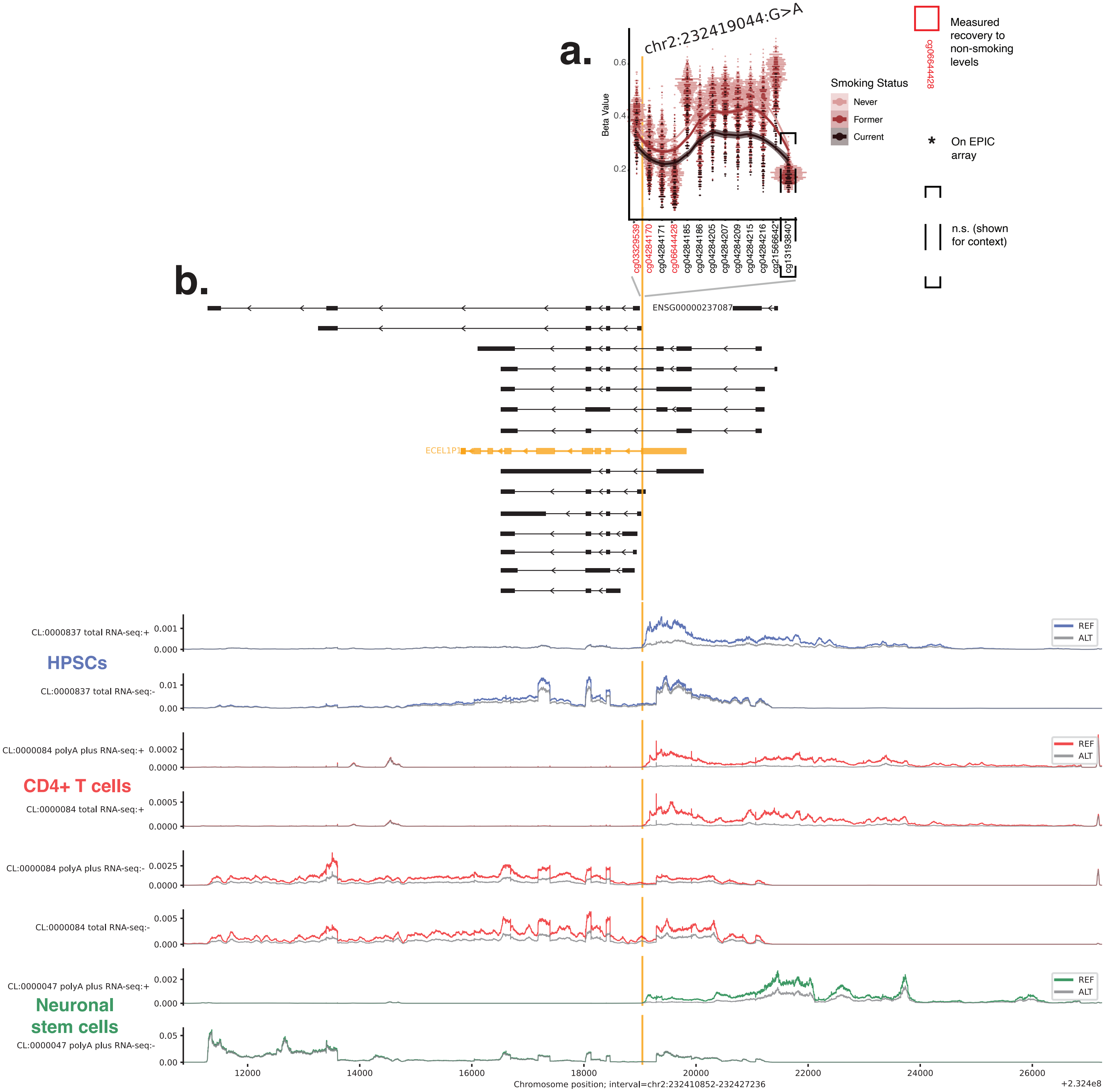

Figure S1: Cell-type-specific effects of DMR-overlapping variant rs79466634 on lncRNA ENSG00000237087 across cell type lineages predicted by AlphaGenome: [a] Beta values for never, former and current smokers in CpGs at *ECEL1P1*. These DMPs form a DMR, including novel MSA-specific CpG cg04284215, which is the top DNA methylation signal in former smokers. Variant rs79466634 is located between DMR CpGs cg03329539 and cg04284170. n.s. (not significant). **[b]** Gene tracks and comparative RNA-seq tracks for the rs79466634 variant (chr2:232,419,044:G>A) across three human cell lineages: hematopoietic stem cells (HPSCs, CL:0000837; top), CD4+ T cells (CL:0000084; middle), and neuronal stem cells (NSCs, CL:0000047; bottom). RNA-seq read density (normalized counts) is shown for the reference (REF, G allele; grey) and alternate (ALT, A allele; coloured) genotypes. Strand-specific expression profiles reveal a stronger transcriptional effect on the positive (+) strand in HPSCs, where the REF (G) allele is associated with an increase in transcript abundance for the long non-coding RNA ENSG00000237087. Notably, similar regulatory effects are observed in both the HPSC and T-cell lineages, suggesting a potential shared regulatory mechanism. In contrast, NSCs show different profiles, highlighting potential lineage-restricted activity of the underlying regulatory element. Genomic context tracks (top) indicate ENSG00000237087 transcript isoforms (in black) and the neighboring *ECEL1P1* pseudogene (in orange). The vertical orange line indicates the position of the candidate causal variant, rs79466634, localized within the bivalent enhancer/TSS flanking region of the affected lncRNA. Interval shown: chr2:232,410,852– 232,427,236 (hg38).
